# Probing the role of perception in fear generalization

**DOI:** 10.1038/s41598-019-46176-x

**Published:** 2019-07-11

**Authors:** J. Zaman, D. Struyf, E. Ceulemans, T. Beckers, B. Vervliet

**Affiliations:** 1 Center for the Psychology of Learning and Experimental Psychopathology, Faculty of Psychology and Educational Sciences, KU Leuven, Tiensestraat 102, Box 3712, 3000 Leuven, Belgium; 2 Health Psychology, Faculty of Psychology and Educational Sciences, KU Leuven, Tiensestraat 102, Box 3726, 3000 Leuven, Belgium; 3 Quantitative Psychology and Individual Differences Research Unit, KU Leuven, Tiensestraat 102, box 3731, Leuven, Belgium; 4 Leuven Brain Institute, KU Leuven, Tiensestraat 102, Box 3712, 3000 Leuven, Belgium; 5 Laboratory for Biological Psychology, Faculty of Psychology and Educational Sciences, KU Leuven, Tiensestraat 102, Box 3714, 3000 Leuven, Belgium

**Keywords:** Human behaviour, Psychology

## Abstract

Behavior in novel situations is guided by similarities to previous experiences, a phenomenon known as generalization. Despite the widespread influence of generalization on healthy and pathological behavior, insight into the underlying mechanisms is lacking. It remains unclear whether a failure to notice situational changes contributes to the generalization of learned behavior. We combined a fear conditioning and generalization procedure with a perceptual decision task in humans and found that a failure to perceive a novel stimulus as different from the initial fear-evoking stimulus was associated with increased conditioned responding. These findings demonstrate the potential of a perception-centered approach to better understand (pathological) behavior and its underlying mechanism and are a promising avenue for the development of refined generalization protocols.

## Introduction

The ability to apply knowledge acquired in one situation to a novel one is key to adaptive behavior in changing environments. Numerous examples across species and behaviors illustrate that such transfer is an ubiquitous phenomenon, with a bell-shaped gradient describing the inverse relationship between response strength and stimulus similarity^1^. Yet the mechanisms that enable this capacity remain an issue of debate^2–4^. The bulk of generalization research uses conditioning procedures where a stimulus (conditioned stimulus, CS) starts to elicit a response (conditioned response, CR), such as fear or avoidance, after it has been linked to a motivational stimulus (unconditioned stimulus, US, e.g., pain). During a test phase, the extent to which novel test stimuli (generalization stimuli, GS) elicit the CR is measured. Typically, the strength or probability of a CR will decrease as a function of physical dissimilarity between the GS and the CS^1,5^, resulting in a bell-shaped gradient peaking around the location of the CS. The shape of the gradient has been used as an argument for generalization as a decision process that occurs when stimulus differences are perceived (i.e., degree of similarity between a conditioned stimulus, CS, and a novel *generalization* stimulus, GS). Recently, this assumption has been challenged by a perceptual account that triggered a renewed interest in the role of perception in this context^2,4,6,7^. It has been argued and demonstrated that stimulus *misidentification*s contribute to generalization gradients^2,4,6,7^. The authors demonstrated that, in the most extreme scenario, a bell-shaped gradient could theoretically merely result from the combination of a binary response strategy, where a GS will only elicit the learned response when being misidentified as the CS, and a probability gradient that a GS is misidentified as the initially fear-evoking stimulus^4^.

As most generalization studies comprised animal work^1^, distinguishing perceptual mechanisms from decision processes proved challenging in the past due to difficulties to assess perception. Moreover, the scientific tradition to interpret behavior within the objective reality in which it occurs – a heritage from early-day behaviorists in their attempt to uncover universal laws of behavior – has rendered generalization researchers unable from addressing the abovementioned issue despite recent advances in related fields. For example, the same learning processes that govern behavior also affect and shape perception^8,9^. After a conditioning procedure, a wider range of tones are misidentified as the initial reinforced tone (CS+) compared to a control tone, and such misidentifications have recently been shown to affect the strength of conditioned responding^6^. Here, we applied these recent advances to test whether problems in stimulus identification contribute to the bell-shaped generalization curve.

In a human fear conditioning procedure, we tested the extent of fear generalization after an acquisition phase, in which a circle of a given size (the CS) was followed by a painful electrocutaneous stimulus (the unconditioned stimulus, US) on 50% of its presentations. During the generalization phase, on each trial one of seven circles of different sizes was presented (6 generalization stimuli, GS, and the original CS). Subjects indicated whether or not the presented stimulus was “the same as the one presented during the previous phase or not” (perceptual categorization task), after which US-expectancy ratings and startle eyeblink reflexes were recorded. In a first control group, the categorization task was omitted (NO CAT group) in order to assess the effects of explicit categorization on the shape of the generalization gradient. In a second control group (NO FEAR group), the aversive US was replaced by a non-aversive visual stimulus in order to compare the effects of aversive versus non-aversive learning on perceptual categorization and generalization.

## Method

### Participants

In the EXP group, three of the forty-three healthy volunteers who participated were excluded in the final analyses due to technical problems [26 females, mean age: 22.08 (4.46 SD)]. In the NO CAT control group, twenty-one healthy volunteers participated and twenty were included in the final sample [due to technical problems; 11 females, mean age: 21.95 (7.50 SD)]. In the NO FEAR control group, 23 healthy volunteers participated [23 females, mean age: 25.00 (10.60 SD)]. Group assignment was counterbalanced across test days. Exclusion criteria based on self-report were (1) a history of cardiac, breathing or cardiovascular disorders, neurological disorders, chronic pain, psychiatric disorders (2) pregnancy, hearing difficulties, acute pain, use of recreational drugs, ongoing recovering from severe trauma, advice from general practitioner to avoid stress, any type of electronic implant (e.g., pacemaker). Volunteers were recruited through local advertisement boards and were paid 15 euros. The study was approved by the social and societal ethics committee (SMEC) of the KU Leuven, Belgium (G-2016-10-641). All study methods were performed in accordance with SMEC guidelines and regulations. All participants provided their written informed consent.

## Materials

### Electrical stimulation

The stimulus was applied at the dorsal end of the ulna at the wrist of the non-dominant hand by a commercially available electrocutaneous stimulation device (Constant Current Stimulator, model DS7; Digitimer©, Hertfordshire, UK) delivering a 2 ms monopolar square waveform pulse via two surface electrodes (V91-01, 8 mm, Coulbourn©) filled with K–Y gel (Johnson & Johnson, New Brunswick, NJ). Stimulation levels were determined for each individual using the Ascending Methods of Limits approach^10^. The stimulation intensity was gradually increased until a score of 8 was reached on a visual analog scale (VAS, 0 = *no sensation, 10* = *extreme, intolerable pain*) or participants indicated they could not tolerate a higher intensity. Mean intensity for the US was 28.23 mA (14.21 SD) in the EXP group and 21.6 mA (9.34 SD) in the NO CAT group. There was no difference in US intensity between both groups [*t*(58) = 1.884, *p* = 0.065]. After the 2^nd^ generalization block, the US was recalibrated. In the NO FEAR group, no electrodes were attached and the calibration phase was skipped. Instead, a picture of a graphical representation of lightning bolt was used as the US.

### Visual stimuli

In total 7 circles (varying diameters from 7.37 to 11.94 cm with steps of 0.762 cm) were created as white lines against a black background, similar to^6,11^.

### Eyeblink startle responses

A frequently adopted psychophysiological index of covert defensive mobilization is the startle reflex, as the strength of its amplitude is greater when the subcortical defensive fear network is activated^12,13^. Startle eyeblink responding was triggered by a 50-millisecond burst of white noise (called the startle probe) with a peak of 105 dB presented binaurally through headphones (Sennheiser, HD418). Orbicularis oculi electromyographic activity (EMG) was recorded using three disposable trimmed Ag/AgCI electrodes (H124SG, 24 mm, Covidien©), according to the guidelines of Blumenthal and colleagues^14^. The skin was abraded with a mild abrasive cream (Inecto). The raw signal was amplified (v75-04, Coulbourn isolated bioamplifier©) with a 13 Hz high pass and 500 Hz low pass bandpass filter and rectified and smoothed with a time constant of 20 ms (V76-24, Coulbourn Integrator©). EMG sampled at 1000 Hz was recorded from 200 ms prior to probe onset until 800 ms after probe onset. Startle eyeblink amplitudes were calculated by subtracting the mean baseline value (0–20 ms after probe onset) from the peak value found in the 21–175 ms time window after the startle probe onset. To reduce inter-individual variation, startle amplitudes were transformed into T-scores and differences scores from the mean ITI startle amplitude per participant were calculated. Data were visually inspected (offline) for artifacts and rejected if necessary. Rejection criteria were spontaneous blinks in the 200 ms interval preceding probe onset or excessive noise in the EMG signal that prevented a clear differentiation between the baseline signal and eyeblinks. Startle data of 16 participants were excluded due to excessive noise [EXP group: n = 1, NO CAT group: n = 5, NO FEAR group: n = 10]. If more than 50% of the startle trials were non-responses, the participant was deemed a non-responder and omitted from analyses (EXP group: n = 10, NO FEAR group: n = 3, NO CAT group: n = 3).

### Protocol

**Figure 1:**
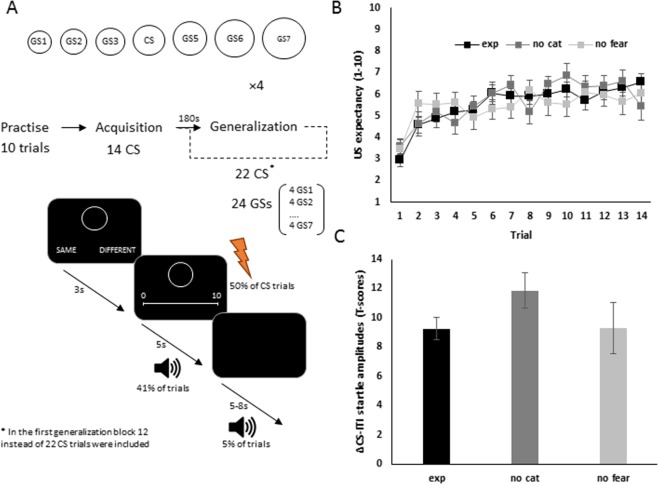
(**A**) Overview of the experimental protocol. Upper panel: Circles of increasing sizes were used as generalization stimuli, the middle circle was used as the conditioned stimulus. Middle panel: Schematic representation of the experimental design. Lower panel: Trial flow during the generalization phase. A circle was presented and participants had to categorize the stimulus as same or different as the circle presented during the acquisition phase. Next, US expectancy was recorded. In case of a reinforced CS trial, a painful electrocutaneous stimulus was presented after 8 seconds. **(B)** Mean US expectancy data across acquisition trials for the different groups. **(C)** Mean difference in CS startle amplitudes during acquisition and overall ITI startle amplitudes. Error bars represent standard errors.

Participants were seated in an adjustable chair at approximately 0.5 m in front of a computer screen. In order to familiarize the participants with the protocol, a practice block of 10 trials was included. Trial structure was identical compared to a generalization trial apart that: one of two squares was presented (instead of circles) which participants had to categorize as same or different (one square was presented prior to the discrimination task) and that a startle probe was presented every trial. Next, during the acquisition phase 14 CS trials were presented. On each trial, the CS was presented and remained visible for 8 seconds. Three seconds after CS onset a VAS appeared for 5 seconds on which participants rated US expectancy (1 = no US, 10 = certain US). At the end of a trial, the circle and VAS disappeared. In case of reinforced trial (50% of the CS trials), the US was presented. In the NO FEAR group the US was a picture of a graphical representation of lightning bolt. Trials were separated by a variable intertrial interval (ITI) (5–8 s). A startle probe was presented in the 4–7 seconds interval post circle onset (in 41% of the trials) or during the ITI (seconds 4–7, in 5% of the trials). The generalization phase comprised four blocks. The first generalization block consisted of 12 CS trials and 24 GS trials (4 per GS). The remaining three blocks, each comprising 22 CS trials and 24 GS trials (4 per GS), started with 10 consecutive CS trials. On every trial during the generalization phase, one of the 7 circles appeared in the middle of the screen with two response options underneath the circle (same vs. different) (see Fig. 1A). Participants indicated within 3 seconds whether they presented stimulus was identical to the stimulus (CS) of the acquisition phase or not (using a mouse click). The cursor’s position was reset every trial at an equal distance between both response alternatives. In the NO CAT group the two-forced choice response options did not appear. While the circle remained on-screen, response options disappeared after 3 seconds and a VAS appeared for 5 seconds on which participants rated US expectancy (1 = no US, 10 = certain US). At the end of a trial, the circle and VAS disappeared. In case of a reinforced trial (50% of the CS trials), the US was presented. When participants failed to respond (perceptual categorization and/or US expectancy rating) within the provided time this was registered as missing value (less the 5% of the data). Trials were presented pseudorandomly with the restrictions: of no more than two consecutive trials with the same circle and no more than 1 consecutive startle trial or a reinforced trial. Blocks were separated by a 3 min break.

### Data analyses

All data is publicly available at the Open Science Framework (osf.io/t8u92). Categorization data was transformed to the probability of being categorized as CS (‘same’ response) per stimulus, resulting in a probability distribution across the stimulus dimension, and were analyzed with a marginal model including Stimulus (continuous: ranging from 0–6) and Stimulus^2^ (to model the bell-shaped gradient) as fixed effects and a repeated measures compound symmetry covariance structure. In a second step, group differences were investigated through the inclusion of an additional factor Group (EXP/NO FEAR) and its interaction with Stimulus and Stimulus^2^.

**Figure 2:**
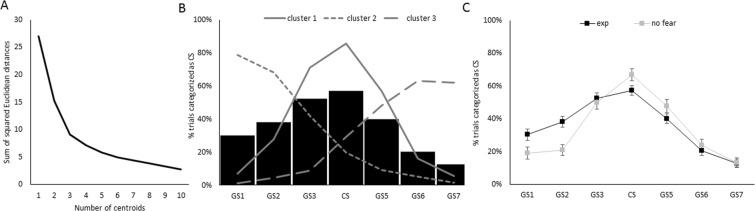
(**A**) Output of the cluster analyses: the sum of the squared Euclidean distances for the different number of centroids. **(B)** Percentage of trials during the generalization phase on which the presented stimulus was identified as the CS in the EXP group (bars). The grey lines are the three identified clusters within the EXP group. **(C)** Percentage of trials during the generalization phase on which the presented stimulus was identified as the CS in the EXP group and NO FEAR group. Error bars represent standard errors.

Explorative, a cluster analysis using the k-means algorithm, as implemented in MATLAB^15^, was used to identify the number of distinct clusters within the calculated probability distributions across the stimulus dimension. A sample size of 40 is sufficient given benchmarking studies on cluster procedures^16^. The algorithm identifies clusters through iterative minimization of the sum of point-to-centroid distances (using the squared Euclidean distance measure). Each subject is allocated to the cluster for which squared Euclidean distance is minimal. The maximum number of centroids was set to 10. 10000 runs of the algorithm with different random initialization of the centroid matrix were performed^15^ to prevent ending in a local optimum. The run with the lowest sum of squared Euclidean distances was retained. The solution with three centroids was preferred as more centroids led to an asymptote (see Fig. 2A).

US expectancy data were analyzed separately for the acquisition and generalization phase per group using mixed models with a random intercept. The model for the acquisition data included Trial (continues) as a fixed effect. In a second step, group differences were investigated through the inclusion of an additional factor Group (EXP/NO FEAR/NO CAT) and its interaction with Trial. For the generalization data, three different models were created. Model 1 included Trial, Stimulus, and Stimulus^2^. In model 2, the categorical variable Categorization (same vs. different), and its interaction with Stimulus and Stimulus^2^ were included. In a final model, group differences were explored (Stimulus, Stimulus^2^, Trial, Group, Group × Stimulus, and Group × Stimulus^2^).

**Figure 3:**
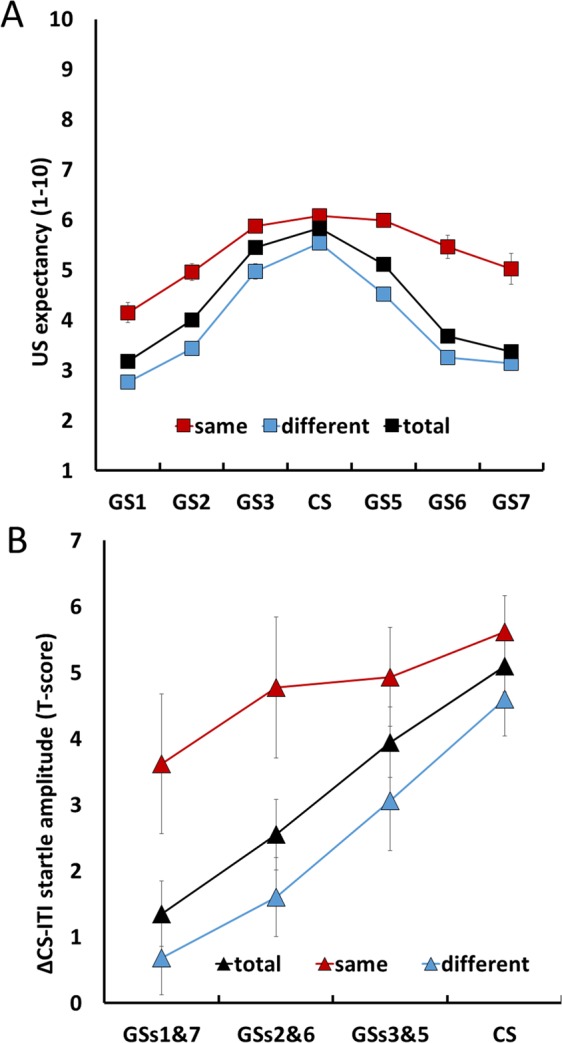
(**A**) US expectancy across stimuli for the EXP group, with (trials on which the stimulus was categorized as CS = same; trials on which the stimulus was categorized as different stimulus = different) and without accounting for CS categorizations (all trials = total). **(B)** Startle amplitudes across the different stimuli for the EXP group, with and without accounting for CS categorizations. Error bars represent standard errors.

Startle amplitudes, converted to T-scores to control for interindividual differences, are expressed as differences scores from ITI startle amplitudes. In order to have sufficient data points for subsequent analyses, GSs on opposite sides of the CS were merged (GS1 & GS7, GS2 & GS6, and GS3 & GS5) (see Fig. 3B). In analogy to the US expectancy analyses, three models were tested. Model 1 comprised Trial and Stimulus_merged_. In model 2, Categorization and its interaction with Stimulus_merged_ were included. In a final model, group differences were explored (Stimulus_merged_, Trial, Group, Group × Stimulus_merged_). All models had a random intercept to account for the repeated measures nature of the data. Post hoc testing was done using the adjusted Bonferroni correction. All analyses were conducted using SPSS 20.

## Results

### Acquisition

#### US expectancy

All three groups learned the association between the CS and the US as US expectancy increased across acquisition trials [EXP group - Trial effect: *F*(1,512.390) = 84.003, *p* < 0.001; NO FEAR group - Trial effect: *F*(1,293.077) = 13.112, *p* < 0.001; NO CAT group - Trial effect: *F*(1,272) = 27.882, *p* < 0.001]. The Group × Trial interaction reached significance [*F*(2,1077.401) = 3.117, *p* = 0.045]. Explorative analyses revealed a larger slope in the EXP group compared to the NO FEAR group [*F*(1,805.430) = 6.513, *p* = 0.011], whereas there was no difference in slope between the NO CAT and the EXP group [*F*(1,784.323) = 0.300, *p* = 0.584]. There were no differences in US expectancy at the last trial of acquisition between the three groups [*F*(2,81) = 1.361, *p* = 0.262] (see Fig. 1B).

#### Startle eye blink responses

In all three groups startle amplitudes were significant larger during the CS compared to ITI amplitudes [EXP group: *t*(34.405) = 10.564, *p* < 0.001; NO FEAR group: *t*(10.503) = 4.019, *p* = 0.002; NO CAT group: *t*(15.231) = 8.585, *p* < 0.001] with no difference between groups [*F*(2,61.359) = 1.050, *p* = 0.356] (see Fig. 1C).

### Generalization

#### Perceptual categorizations

Analyses of the perceptual categorizations in the EXP group (probability of CS categorization per stimulus) revealed the typical bell-shaped gradient at the group level [Stimulus effect: *F*(1,250) = 33.059, *p* < 0.001; Stimulus^2^ effect: *F*(1,250) = 44.278, *p* < 0.001]. Overall in 32% [SD = 32,7%] of the trials where a GS was presented, a perceptual error was made, and the probability of misidentification increased as a GS approached the size of the CS circle [Stimulus effect: *F*(1,208) = 19.593, *p* < 0.001 and Stimulus^2^ effect: *F*(1,208) = 26.991, *p* < 0.001] (CS data removed from the model). Explorative analyses revealed an asymmetry as GSs on the left side of the CS (smaller circles) were more often misidentified as the CS compared to GSs on the right side (larger circles) [*F*(1,207) = 14.074, *p* < 0.001] (Marginal model with side (left vs right) and Corresponding GS (1&7 vs 2&6 vs 3&6) as within-subjects factor and a repeated measures effect with compound symmetry as covariance structure). Surprisingly, the stimulus used as CS was only correctly identified on 56% (SD = 35,4%) of the trials. Final, we found that in the EXP group compared to the NO FEAR group overall more stimuli were classified as the CS [Group effect: *F*(1,418.909) = 4.821, *p* = 0.029] but no difference regarding the perceptual gradient between both groups [Group × Stimulus effect: F(1,386) = 2.986, *p* = 0.085; Group × Stimulus^2^ effect: *F*(1,386) = 1.867, *p* = 0.173]. The results of the NO FEAR group can be found in the SI.

Exploratory cluster analyses (k-means algorithm, MATLAB©) revealed three distinct patterns across participants’ categorization data (see Fig. 2B): either CS categorizations were mainly centered around the CS (cluster 1: 52.5% of the participants, n = 21), or they were located at the smallest (cluster 2: 32.5% of the participants, n = 13), or the largest circles (cluster 3: 15% of the participants, n = 6) (similar clusters were identified in the NO FEAR group, see SI for more details).

#### US expectancy

Analyses of US expectancy in the EXP group regardless of the perceptual categorizations revealed the typical bell-shaped gradient [Stimulus effect: *F*(1,6579.046) = 821.908, *p* < 0.001; Stimulus^2^ effect: *F*(1,6579.048) = 917.287, *p* < 0.001]. Furthermore, we found a significant trial effect [*F*(1,6579.062) = 101.387, *p* < 0.001], as US expectancy ratings slightly increased across trials {*β* = 0.013 (0.001), 95% CI [0.011 0.016]}. Explorative analyses revealed that US expectancy ratings were higher for GSs on the left side of the CS compared to GS on the right side of the GS [µ_left_side_ = 4.239, µ_right_side_ = 4.096; *F*(1,3794) = 4.60, *p* = 0.032]. In model 2, the role of perception was investigated through inclusion of the perceptual categorizations in the model. We found that the categorization of a stimulus as CS led to higher US expectancy [Categorization effect: *F*(1,6592.893) = 31.005, *p* < 0.001] but did not affect the shape of the gradient [Stimulus × Categorization effect: *F*(1,6603.168) = 0.839, *p* = 0.36; Stimulus^2^ × Categorization effect: *F*(1,6600.771) = 0.503, *p* = 0.48] (Fig. 2A).

**Figure 4:**
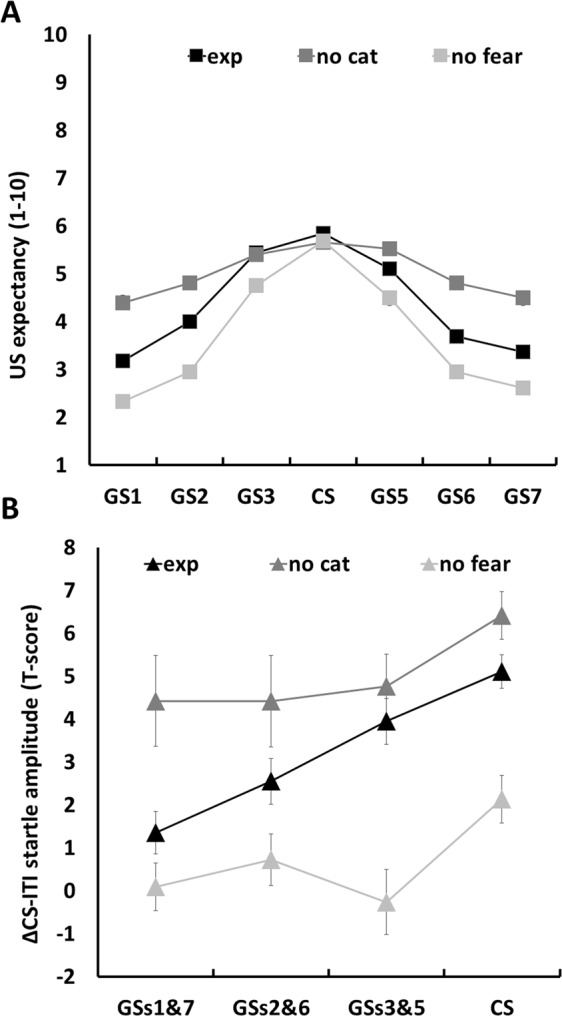
(**A**) US expectancy across stimuli for the different groups. (**B**) Startle amplitudes across stimuli for the different groups. Error bars denote standard errors.

Finally, we tested for group differences between the EXP group and the two control conditions. We found that all groups demonstrated a gradient in the strength of elicited responses across stimuli (see Fig. 4A). The highest overall US-expectancy rating was found in the NO CAT group, and the lowest ratings in the NO FEAR group [main effect of group: *F*(2,104.165) = 17.238, *p* < 0.001]. Furthermore, the inclusion of a perceptual categorization task led to a steeper gradient [(NO CAT vs. EXP) × Stimulus effect: *F*(1,10038.013) = 113.094, *p* < 0.001; (NO CAT vs. EXP) × Stimulus^2^ effect: *F*(1,10038.014) = 135.059, *p* < 0.001], whereas a non-aversive US further steepened the gradient [(NO FEAR vs. EXP) × Stimulus effect: *F*(1,10294.191) = 32.570, *p* < 0.001; (NO FEAR vs. EXP) × Stimulus^2^ effect: *F*(1,10294.195) = 31.559, *p* < 0.001].

#### Startle eye blink responses

In the EXP group, startle amplitudes increased as GSs approach the CS [Merged Stimulus effect: *F*(1,1898.712) = 128.904, *p* < 0.001]. Post hoc testing revealed, that all GS pairs [apart from GS1&7: *t*(48.458) = 1.600*, p* = 0.12] and the CS elicited significantly enhanced startle potentiation compared to the ITI [GS2&6: *t*(49.106) = 4.753*, p* = 0.012; GS3&5: *t*(49.106) = 4.753*, p* < 0.001; CS: *t*(37.052) = 6.494*, p* < 0.001]. Furthermore, the categorization of a stimulus as the CS increased startle amplitudes [Categorization effect: *F*(2,1920.338) = 8.874, *p* = 0.003] but did not affect the shape of the gradient [Merged Stimulus × Categorization effect: *F*(1,1907.638) = 2.351, *p* = 0.13].

Finally, we investigated group differences through inclusion of Group and its interaction with Merged Stimulus. The main effect of Group was not significant [*F*(1,71.879) = 1.834, *p* = 0.17], whereas differences between groups regarding the shape of the gradient were found [Merged Stimulus × Group effects: *F*(1,3322.669) = 3.365, *p* = 0.035]. Post hoc testing revealed a gradient [merged stimulus effect: *F*(1,833) = 11.504, *p* < 0.001] in the NO FEAR group that was flatter compared to the gradient of the EXP group [(EXP vs. NO FEAR) × Merged Stimulus effect: *F*(1,2500.232) = 5.127, *p* = 0.024]. None of the startle amplitudes differed from ITI startle amplitudes in the NO Fear group (all *p*’s > 0.21). In the NO CAT group, all GS pairs and the CS elicited significantly higher startle amplitudes compared to the ITI (one-tailed t-tests from 0, all *p*’s ≤ 0.004) and increased across stimuli [merged stimulus effect: *F*(1,492) = 8.324, *p* = 0.004] similar to the EXP group [*F*(1,2813.538) = 3.511, *p* = 0.061] (Fig. 4B).

## Discussion

In this study, we combined a generalization protocol with a perceptual categorization task to investigate whether generalization gradients at least partially relate to perceptual errors. We found that the probability gradient with which a stimulus is (incorrectly) identified as the conditioned fear stimulus (i.e., a *perceptual* gradient) strongly resembles the typical shape of a fear generalization gradient. Stimuli perceived as the conditioned fear stimulus were associated with a higher fear response compared to when the same stimuli were perceived as different.

We demonstrated that during the assessment of generalized responding across a physical dimension stimuli are often mistaken for the initially trained stimulus and that these perceptual errors were associated with stronger fear responses both for explicit and implicit measures. These findings suggest a close relationship between perceptual errors and generalized fear responses across a physical continuum. At the same time, they demonstrate that generalization is also driven by other processes than perceptual errors as gradients in responding were still found after accounting for perceptual errors. The observed decrement in responding across stimuli within each perceptual category seems difficult to reconcile with a mere perceptual account of generalization where identical percepts are hypothesized to result in similar strengths of responding. Future studies should investigate to what extent other aspects such as decision certainty or perceived difference may further explain these findings. For instance, the perception of a stimulus as CS can be based on little or a lot of evidence. As uncertainty increases, the assumed impact on conditioned responding can be expected to wane. Similarly, due to the binary nature of the task, the categorization of a stimulus as different from the CS (i.e., GS percept) can comprise a range of perceived difference. Furthermore, our design does not enable us to assess whether perception determines the strength of fear responding or vice versa. In contrast with a perceptual account, one could argue that fear affects the perceptual system such that the perception of fear-evoking stimuli is favored^17^. In line with the latter hypothesis, we did find more CS percepts in the EXP compared to the NO FEAR group. However, we also found similar perceptual gradients and effects of perception on conditioned responding in the NO FEAR group, suggesting that fear is not a requisite to observe these effects.

Interindividual differences notwithstanding, considerable proportions of the presented stimuli during the generalization phase were misidentified. The probability with which a stimulus was identified as the conditioned fear stimulus peaked at the CS location and decreased as a function of physical CS-GS distance. This bell-shaped perceptual gradient bares a strong resemblance to the common generalization gradient. Interestingly, we found large variations between individuals regarding perceptual accuracy. The overall perceptual gradient emerged from a combination of three predominant types of categorization patterns: participants with CS categorizations that were centered around the CS (cluster 1), or those where it shifted towards the extreme GSs (either the largest or the smallest circles) (cluster 2 and 3). A recent study identified similar clusters of categorization patterns in a larger sample^7^. Reasons for the perceptual biases (cluster 2 and 3) remain speculative at present but may relate to differences in spatial tuning at primary visual brain regions^18^, biased perceptual decision-making, be indicative of a memory bias^19^ or could reflect differences in the interpretation of the task instruction. Future research should investigate the role of these distinct explanations in more depth. Interestingly, patterns of perceptual accuracy have been linked to different experienced stimulus-outcome contingencies across the generalization phase, with identical experienced contingencies for CS-percepts and GS-percepts^7^ in certain cases (see also SI analyses). Furthermore, in those previous studies, the effect of perception on conditioned responding was found to depend on those subjective contingencies^7^ (see also SI analyses), suggesting that perceptual errors may influence conditioned responding directly but also more indirectly. In a recent study, Laufer and colleagues (2016) demonstrated that anxiety patients identified a wider range of stimuli as the CS after a conditioning protocol compared to healthy controls. As such, previous reports on differences in generalization tendencies between anxiety patients and healthy controls^20^ should be interpreted cautiously as they may reflect either difference in perceptual acuity between patients and controls or a cognitive risk-aversion bias^21^.

The influence of verbalized decision-rules on the shape of the generalization gradient^22^ and the observation that generalized responding occurs across a range of distinct objects belonging to the same category^23,24^, demonstrate the role of cognitive processes in generalization. Although various forms of generalization exist, the most studied form is across a continuum of physically similar stimuli^1^. The current findings show the importance of perception, in addition to cognitive processes, in a context of generalization across physically similar stimuli. These results illustrate the limitations of an approach^25^ where behavioral generalization is interpreted without taking into account the perception of the stimuli. At the same time, they demonstrate that generalization is not a pure by-product of perception either, as similarity-based fear gradients were observed even for stimuli that were correctly identified as novel. However, the exclusive focus on conditioned responses in previous research has arguably led researchers to create models of generalization that ignore the potential impact of perceptual stochasticity. Therefore, if such behavioral readouts are considered as potential biomarkers^20^ and treatment indicators^26^, one must ascertain that they are developed in such a way that their discriminative and predictive power is maximized.

Finally, some limitations should be acknowledged. Given the relatively small sample for the startle eyeblink data due to exclusion of non-responders and problematic recordings, replication of the findings seems warranted. In addition, as the use of startle probes has been found to interfere with safety learning^27^, future studies may want to use other psychophysiological indices of fear learning. Second, the explicit perceptual categorization task most likely promoted attention to distinctive stimulus features, thereby affecting the degree of perceived similarity between a GS and the CS, and as such could directly affect the generalization gradient. We indeed found that the explicit categorization of stimuli during fear generalization testing led to a sharpening of the generalization gradient. However, the effect of incorporating a perceptual categorization task on the generalization gradient was limited in that it was found in an explicit measure only (US expectancy but not startle). Final, the NO FEAR group did not differ from the other groups on startle difference scores during acquisition. This might be due to the use of averaged ITI responses as the subtrahend. The majority of startle ITI responses were recorded during the generalization phase. Hence, a positive difference score might merely result from response habituation across the experiment. The lack of startle potentiation relative to ITI amplitudes during the generalization phase does suggest the absence of fear learning in the NO FEAR group.

In sum, we demonstrated that congruent assessment of perception and fear enables an in-depth understanding of the mechanisms underlying transfer of learning. We found that the misperception of a generalization stimulus as the conditioned fear stimulus contributed to the shape of the obtained generalization gradient.

## Supplementary information

supplementary info
