# Supplementary material for: Probing the role of perception in fear generalization
Source: Sci Rep. 2019 Jul 11;9:10026. doi: 10.1038/s41598-019-46176-x (PMC6624264; doi:10.1038/s41598-019-46176-x)
Supplement: Supplementary file 1 — supplementary info [file 41598_2019_46176_MOESM1_ESM.docx]

**Probing the role of perception in fear generalization**

Zaman, J., Struyf, D., Ceulemans, E., Beckers, T., Vervliet, B.

**SUPPLEMENTAL MATERIAL**

**1. US-expectancy (acquisition data) – Mixed model**

| **Acquisition** | **EXP GROUP** | **NO FEAR GROUP** | **NO CAT GROUP** |
| --- | --- | --- | --- |
| Fixed effects | Parameter Estimate (standard error) | Parameter Estimate (standard error) | Parameter Estimate (standard error) |
| Intercept | 4.15(.28)*** | 4.75(.41)*** | 4.41(.34)*** |
| Trial | .19(.02)*** | .10(.03)*** | .16(.03)*** |
| Random effects |  |  |  |
| Variance RI | 1.84 | 2.55 | .93 |
| Residual variance | 3.70 | 3.79 | 4.74 |

RI = Random Intercepts , ^ < .1, * < .05, ** < .01, *** < .001

**2. Perceptual categorizations (Probability of CS responses, generalization data) – Marginal model**

| **Generalization** | **EXP GROUP** | **NO FEAR GROUP** |
| --- | --- | --- |
| Fixed effects | Parameter Estimate (standard error) | Parameter Estimate (standard error) |
| Intercept | .06(.08) | -.24(.10)* |
| Stimulus | .25(.04)*** | .38(.06)*** |
| Stimulus^2^ | -.04(.01)*** | -.05(.01)*** |
| Repeated measures effects |  |  |
| Com. Symm. Diagonal offset | .10 | .10 |
| Com. Symm. covariance | -.003 | -.003 |

Com. Symm. = Compound Symmetry, ^ < .1, * < .05, ** < .01, *** < .001

**Additional analyses – clustering NO FEAR group**

Exploratory cluster analyses (k-means algorithm, MATLAB©) revealed three distinct patterns across participants’ categorization data (see Fig. S1): either CS categorizations were mainly centered around the CS (cluster 1: 70 % of the participants, n = 16), or they were located at the smallest (cluster 2: 13.0 % of the participants, n = 3), or the largest circles (cluster 3: 17 % of the participants, n = 4).


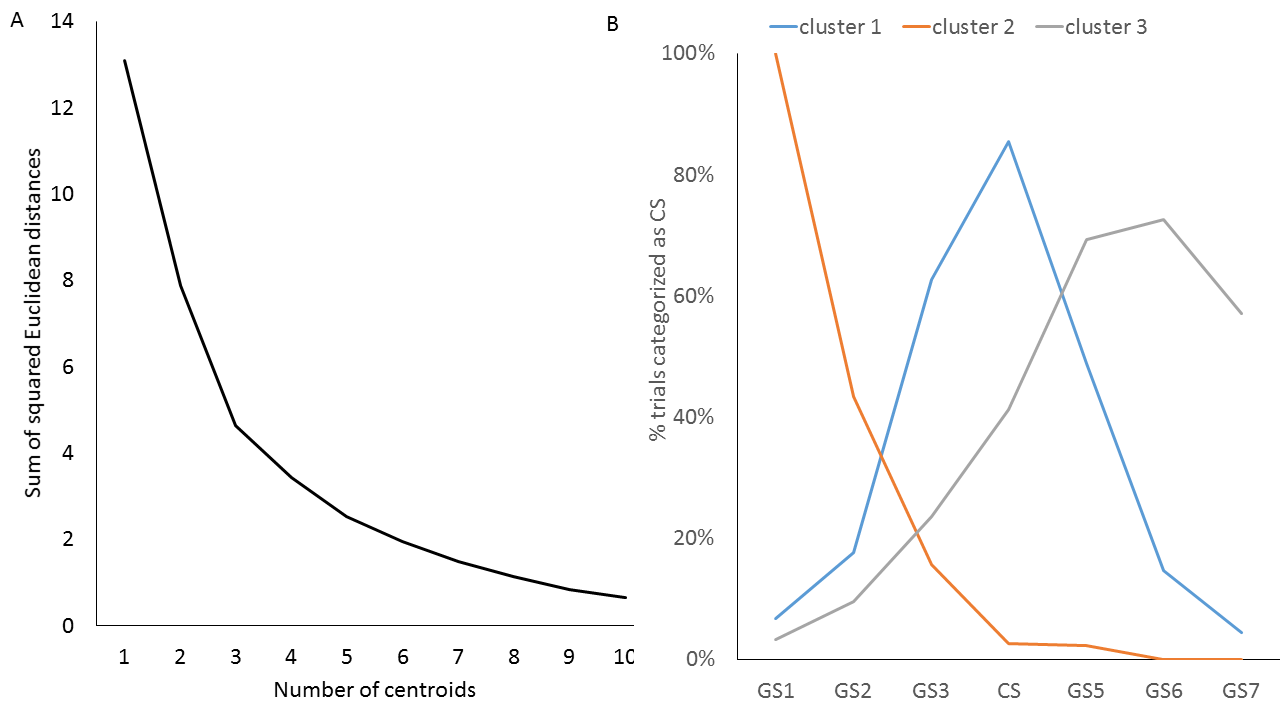


Fig. S1. (A) Output of the cluster analyses of the NO FEAR group: the sum of the squared Euclidean distances for different number of centroids. (B) The three identified clusters within the NO FEAR group.

**3. US-expectancy (generalization data) – Mixed models**

| **EXP GROUP** | **Model 1** | **Model 2** | **Model 3** |
| --- | --- | --- | --- |
| Fixed effects | Parameter Estimate (standard error) | Parameter Estimate (standard error) | Parameter Estimate (standard error) |
| Intercept | 3.00(.24)*** | 2.80(.25)*** | 4.26(0.37)*** |
| trial | .01(.00)*** | .01(.00)*** | .01(0.00)*** |
| Stimulus | 1.51(.05)*** | 1.18(.06)*** | .77(0.09)*** |
| Stimulus^2^ | -.26(.01)*** | -.20(.01)*** | -.15(0.01)*** |
| Categorization |  | .87(.16)*** | -.65(.20)*** |
| Cat. × Stim |  | .10(.11) | .02(.13) |
| Cat. × Stim^2^ |  | .01(.02) | .05(.02)* |
| Cluster |  |  | -2.23(.50)*** |
| Stim × Cluster |  |  | .26(.12)* |
| Stim^2^ × Cluster |  |  | -.02(.02) |
| Cat. × Cluster |  |  | 4.39(.34)*** |
| Cat. × Cluster × Stim |  |  | -1.01(.24)*** |
| Cat. × Cluster × Stim^2^ |  |  | .12(.04)** |
| Random effects |  |  |  |
| Variance RI | 2.14 | 2.21 | 2.18 |
| Residual variance | 4.08 | 3.75 | 3.40 |

RI = Random Intercepts , ^ < .1, * < .05, ** < .01, *** < .001

| **NOFEAR GROUP** | **Model 1** | **Model 2** |
| --- | --- | --- |
| Fixed effects | Parameter Estimate (standard error) | Parameter Estimate (standard error) |
| Intercept | 1.93(.23)*** | 1.95(0.22)*** |
| trial | .01(.00)*** | 0.01(0.00)*** |
| Stimulus | 1.96(.08)*** | 1.06(0.09)*** |
| Stimulus^2^ | -.32(.01)*** | -0.18(0.02)*** |
| Categorization |  | 1.20(0.27)*** |
| Cat.. × Stim |  | 0.75(0.18)*** |
| Cat.. × Stim^2^ |  | -0.11(0.03)*** |
| Random effects |  |  |
| Variance RI | 0.96 | 0.83 |
| Residual variance | 5.49 | 4.61 |

RI = Random Intercepts , ^ < .1, * < .05, ** < .01, *** < .001

| **NOCAT GROUP** | **Model 1** |
| --- | --- |
| Fixed effects | Parameter Estimate (standard error) |
| Intercept | 4.28(.21)*** |
| trial | .01(.00)*** |
| Stimulus | .73(.09)*** |
| Stimulus^2^ | -.12(.01)*** |
| Random effects |  |
| Variance RI | .63 |
| Residual variance | 5.76 |

RI = Random Intercepts , ^ < .1, * < .05, ** < .01, *** < .001

**3.1. Post hoc analyses: US expectancy gradients per cluster**

Inspection of Fig. S2, reveals a peak in US expectancy ratings at the location of the CS and its neighboring GSs in each of the clusters. In Cluster 1 (n = 21) US expectancy rating at CS were significant higher compared to ratings at GS3 (β = .576, SE = 0.12, p < .001, 95% CI = [0.344 0.81]) and GS5 (β = .913, SE = 0.12, p < .001, 95% CI = [0.680 1.146]). In Cluster 2 (n = 13), the highest US expectancy rating were found at the CS compared to ratings at GS5 (β = .584, SE = 0.147, p < .001, 95% CI = [0.295 0.873]) but not GS3 GS5 ((β = .108, SE = 0.147, p > .9, 95% CI = [-0.181 0.397]). For Cluster 3 (n = 6), results should be interpreted with caution due to the low number of subjects allocated to this cluster. US expectancy ratings did not differ between the CS and its adjacent GSs (*p*’s > .132) but were significant larger compared to GS2 (β = 1.091, SE = 0.226, p < .001, 95% CI = [0.647 1.534]) and GS6 (β = 0.539, SE = 0.226, p = .017, 95% CI = [0.095 0.982]).

Fig. S2. US expectancy per cluster. Error bars denote standard errors.

**3.2. Exploratory analyses investigating effects of Cluster on generalization gradients**

In two explorative analyses the influence of Cluster allocation was investigated. The rational for these analyses was the following: the extent to which perception affects conditioned responding depends on whether people still experienced the CS to be predictive of the US [as demonstrated in^16^]. Given the large variation in correct CS categorizations during the generalization phase, there is reasonable doubt whether this was the case for all participants. We will briefly elaborate this idea: the US is presented during generalization in order to prevent extinction and this presentation does not take into account the perception of the CS (as in all conditioning studies). When the CS is correctly identified, one has the opportunity to learn that the CS remains predictive of the US. If the CS is incorrectly perceived as a *different* stimulus the subsequent presentation of the US will yield another learning experience: not only the CS but also other stimuli predict the US. In order to test this hypothesis we combined clusters 2 and 3 (separate analyses for Cluster 2 and 3 revealed similar gradient in both clusters, see SI) into one group (N = 19) as both were characterized by many perceptual errors on CS trials and compared them to participants of cluster 1 (N = 21) that had overall few perceptual errors on CS trials. In the mixed model for the US expectancy rating the additional factor Cluster_combined and the interaction with Stimulus, Stimulus^2^ and Categorization were included. For the startle data analyses, Cluster_combined and the interaction with Stimulus_merged_ and Categorization were included.

**3.2.1. US expectancy**

We found that the effect of perception was depend on the cluster type [Categorization × Cluster effect: *F*(1,6579.611) = 163.309, *p* < .001]. In participants with few errors on CS trials (cluster 1), the categorization of a stimulus as CS increased US expectancy ratings {*β* = 2.534 (.081), 95% CI [2.38 2.69]} whereas in the other group (cluster 2 and 3) this did not affect US expectancy ratings {*β* = -.11 (.085), 95% CI [-.27 .06]}. Furthermore, the shape of the gradient differed between clusters [Stimulus × Cluster effect: *F*(1,6574.111) = 4.472, *p* = .034; Stimulus^2^ × Cluster effect: *F*(1,6574.701) = 4.986, *p* = .026], with a flatter gradient in those participants that made many errors on CS trials (cluster 2 and 3). Interestingly, overall US expectancy ratings were similar between both groups [Cluster effect: *F*(1,47.518) = .004, *p* = .95]. Furthermore, in this model the perceptual categorization of a stimulus as CS led to different gradients compared to when stimuli were categorized as GS [Stimulus × Categorization effect: *F*(1,6577.768) = 17.492, *p* < .001; Stimulus^2^ × Categorization effect: *F*(1,6576.385) = 32.05, *p* < .001]. This interaction effect furthermore depend upon cluster type [Stimulus × Categorization × Cluster effect: *F*(1,6576.975) = 9.115, *p* = .003; Stimulus^2^ × Categorization × Cluster effect: *F*(1,6578.502) = 18.149, *p* < .001]. As displayed in Fig. S3, participants that made many errors on CS trials (cluster 2 and 3) have similar overlapping flat gradients for the different perceptual categorizations. In participants with few errors on CS trials (cluster 1) high US expectancy ratings were observed when a stimulus was categorized as CS irrespective of the CS-GS distance (Fig. S3). For GS categorizations US expectancy ratings were overall lower and increased as the CS-GS distance decreased.

**3.2.2. Startle eye blink responses**

In contrast to the US-expectancy data, startle amplitudes were not affected by the inclusion of cluster nor by any of its interactions (all *p*’s > .089). As startle amplitudes tend to habituate^17^, we found a significant Trial effect indicating that startle amplitudes decreased across trials [*F*(1,1901.597) = 83.907, *p* < .001].


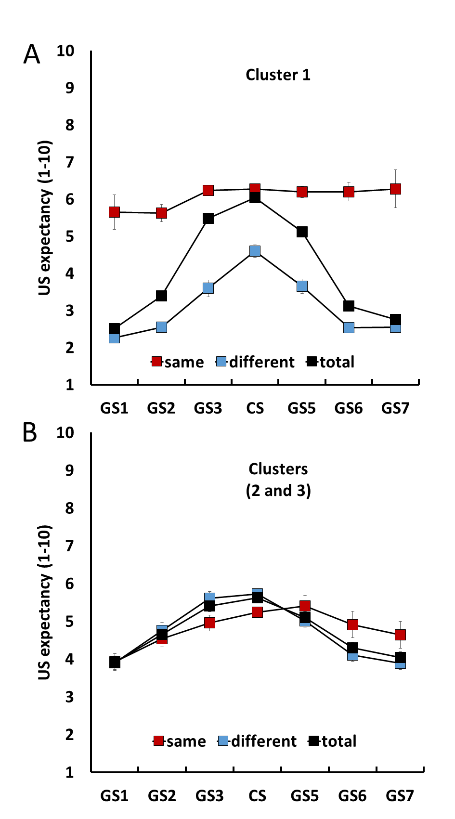


Fig. S3. (A) US expectancy across stimuli for participants in the EXP group that made few errors on CS trials (cluster 1), with (trials on which the stimulus was categorized as CS = same; trials on which the stimulus was categorized as different stimulus = different) and without accounting for CS categorizations (all trials = total). (B) US expectancy across stimuli and for participants with many errors on CS trials (cluster 2 and 3 combined) with and without accounting for CS categorizations. Error bars represent standard errors.

**4. Startle eye blink responses (generalization data) – Mixed models**

| **EXP GROUP** | **Model 1** | **Model 2** | **Model 3** |
| --- | --- | --- | --- |
| Fixed effects | Parameter Estimate (standard error) | Parameter Estimate (standard error) | Parameter Estimate (standard error) |
| Intercept | 1.66(.81)* | .95(.85) | 1.16(1.22) |
| trial | -0.32(.04)*** | -.30(.04)*** | -.31(.04)*** |
| Merged Stimulus | 2.91(.26)*** | 2.94(.30)*** | 2.89(.04)*** |
| Categorization |  | 2.78(.93)** | 3.27(1.12)** |
| Cat. × Merg. Stim. |  | -.70(.45) | -1.12(.61) ^ |
| Cluster |  |  | -.41(1.73) |
| Cat. × Cluster |  |  | -2.26(2.10) |
| Merged Stimulus × Cluster |  |  | -.34(.59) |
| Cat. × Cluster × Merged Stimulus |  |  | .96(1.04) |
| Random effects |  |  |  |
| Variance RI | 14.88 | 14.84 | 15.45 |
| Residual variance | 95.25 | 90.67 | 90.67 |

RI = Random Intercepts , ^ < .1, * < .05, ** < .01, *** < .001

| **NOFEAR GROUP** | **Model 1** | **Model 2** |
| --- | --- | --- |
| Fixed effects | Parameter Estimate (standard error) | Parameter Estimate (standard error) |
| Intercept | .47(1.48) | .67(1.54) |
| trial | -.27(.08)*** | -.289(.08)*** |
| Merged Stimulus | 1.76(.49)*** | 1.36(.60)* |
| Classification |  | .18(2.15) |
| Class. × Merg. Stim. |  | .58(1.02) |
| Random effects |  |  |
| Variance RI | 15.78 | 15.37 |
| Residual variance | 91.64 | 91.73 |

RI = Random Intercepts , ^ < .1, * < .05, ** < .01, *** < .001

| **NOCAT GROUP** | **Model 1** |
| --- | --- |
| Fixed effects | Parameter Estimate (standard error) |
| Intercept | 3.97(1.28)* |
| trial | -.19(.05)*** |
| Merged Stimulus | 1.66(.40)*** |
| Random effects |  |
| Variance RI | 16.48 |
| Residual variance | 95.10 |

RI = Random Intercepts , ^ < .1, * < .05, ** < .01, *** < .001

**5. Experienced Stimulus-Outcome contingencies**

Explorative, two indices were calculated to test how perceptual errors affected experienced CS-US and GS-US contingencies: (1) total number of reinforced CS trials that were correctly perceived as CS relative to the total number of trials in which the stimulus was perceived as the CS. This ratio was used as a proxy for the experienced CS-US contingency; (2) total number of reinforced CS trials that were incorrectly classified as GS relative to the total number of trials in which the stimulus was perceived as a GS. This index was used as a proxy for the experienced GS-US contingency. A one-way ANOVA revealed significant differences between cluster 1 and the other clusters in experienced CS-US and GS-US contingencies [CS-US contingency: *F*(1, 38) = 80.70, *p* < .001; GS-US contingency: *F*(1, 38) = 151.54, *p* < .001]. Post-hoc paired t-test revealed significantly higher CS-US contingencies than GS-US contingencies for Cluster 1 [*t*(20) = 15.51, *p* < .001] (see Fig. S4), whereas in the other participants (Clusters 2 and 3 combined) experienced GS-US contingencies during the generalization phase were higher than experienced CS-US contingencies [*t*(18) = 3.66, *p* = .002].


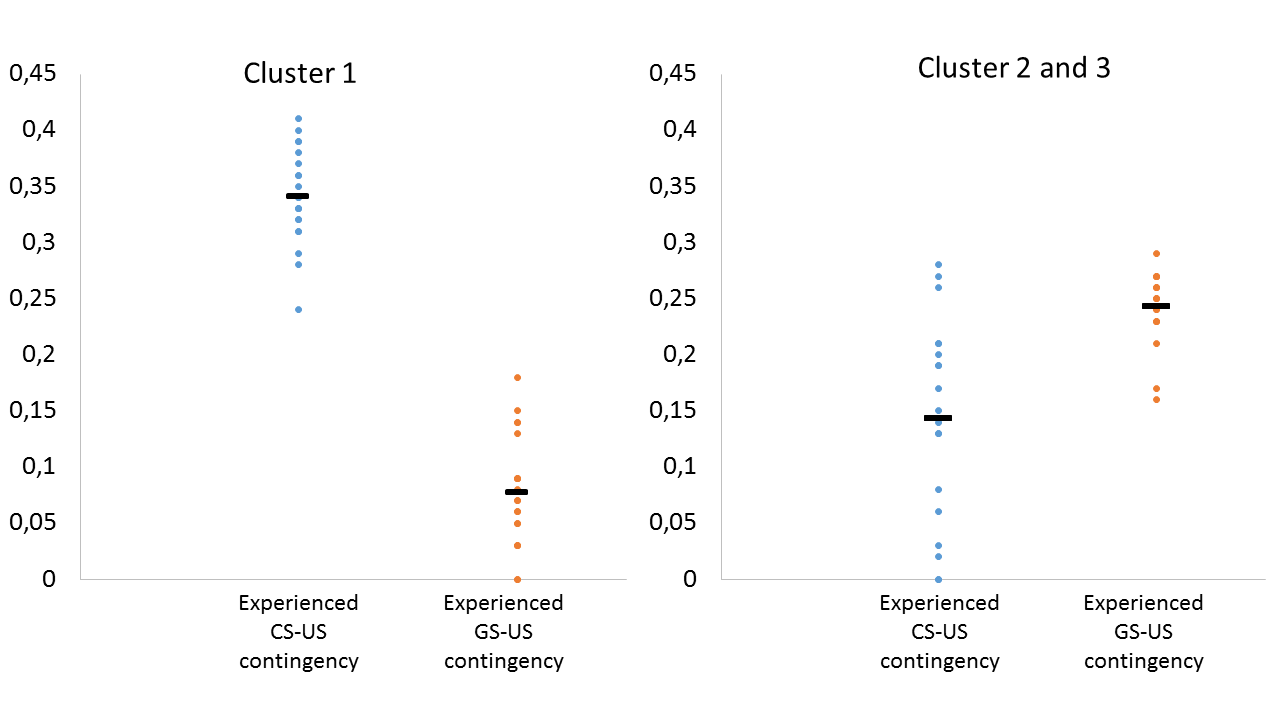


Fig. S4. Experienced stimulus-outcome contingencies for the different clusters.

**6. Gradients between clusters across generalization blocks**

Another explorative analysis was ran on a dataset that only comprised data from the first and the last generalization block, to test whether gradients between clusters diverged throughout the generalization phase. As illustrated in fig. S5, clusters demonstrated relatively similar US expectancy gradients during the first block of the generalization phase, whereas in the last block of the generalization phase distinct gradients emerged between Cluster 1 and the other two clusters. Differences in perceptual errors throughout the generalization phase led to different experienced stimulus-outcome contingencies, gradually producing different gradients. This effect was also reflected in a significant interaction between Cluster, Block and Stimulus (Block (first vs. last) × Cluster (cluster 1 vs Clusters 2 and 3) × Stimulus: *F*(1, 2824) = 22.795, *p* < .001; Block × Cluster × Stimulus^2^: : *F*(1, 2824) = 17.081, *p* < .001).


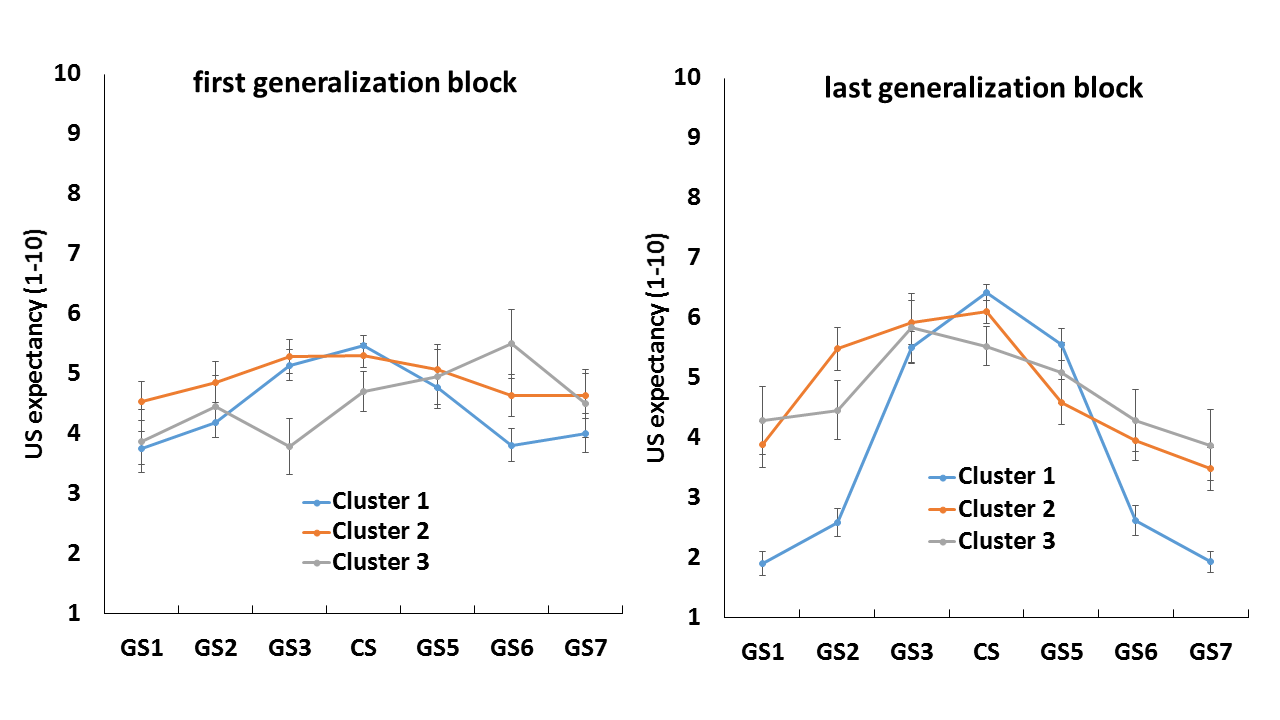


Fig. S5. US expectancy gradients per cluster for the first generalization block (left panel) and the last generalization block (right panel). Error bars denote standard errors.

**7. Impact of perception across generalization blocks**

A final explorative analysis was ran on the dataset that only comprised data from the first and the last generalization block to test whether the impact of perception on response strength evolved differently throughout the generalization phase between the clusters as they experienced different stimulus-outcome contingencies during this phase due to differences in committed perceptual errors. As expected, we found a significant interaction between Block (first vs. last), Categorization (CS percept vs. GS percept) and Cluster (cluster 1 vs. Clusters 2 and 3) [*F*(3, 2828.41) = 59.84, *p* < .001]. Across generalization blocks, US expectancy ratings increased for CS percepts [*F*(1, 2825.75) = 49.11, *p* < .001] and decreased for GS percepts in Cluster 1 [*F*(1, 2826.51) = 63.03, *p* < .001]. In the other clusters, the opposite was found with a decrease in US expectancy ratings for CS percepts [*F*(1, 2829.06) = 12.80, *p* < .001] and an increase in US expectancy for GS percepts across generalization blocks [*F*(1, 2825.37) = 12.72, *p* < .001] (Fig. S6).


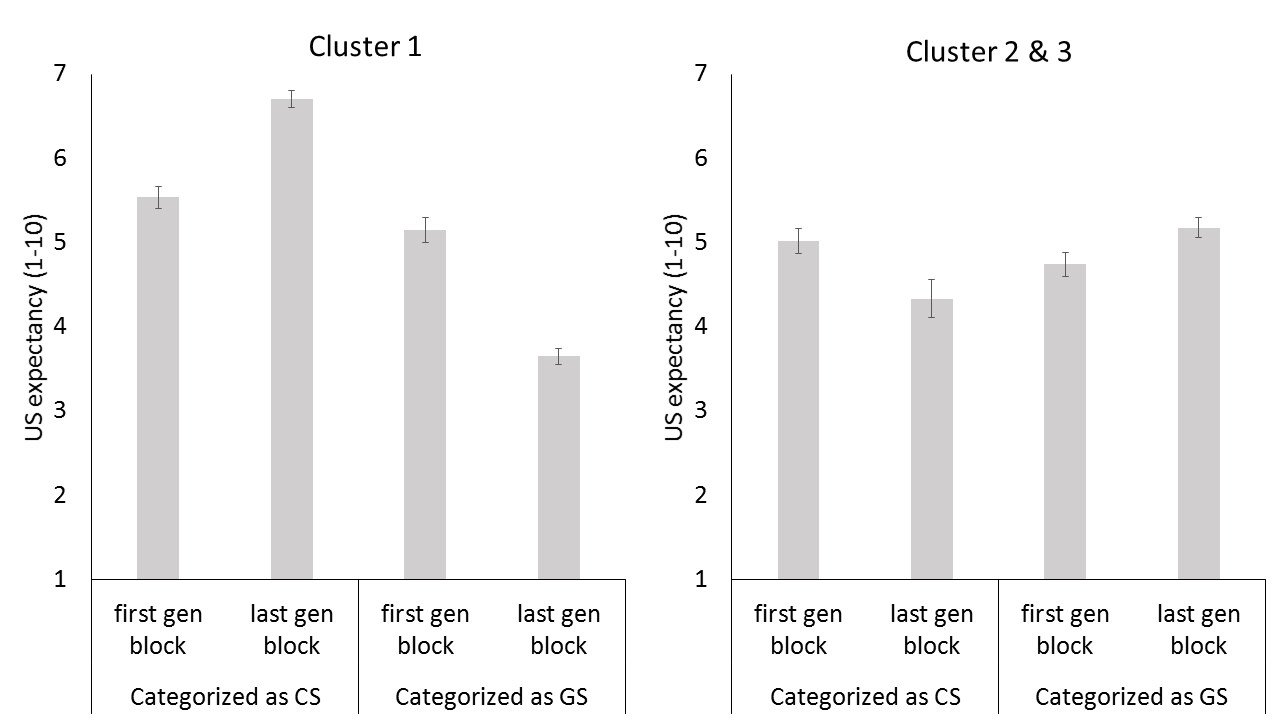


Fig. S6. US expectancy ratings during the first generalization block and the last generalization block per stimulus perception. Error bars denote standard errors

**8. Discussion**

We found that the effect of perception was dependent upon the amount of perceptual errors made during CS trials and that distinct generalization gradients were obtained depending on the distribution of perceptual errors across the stimulus spectrum.

We demonstrated that differences in the amount of perceptual errors on CS trials can yield a different learning experience for the participant compared to the one intended by the experimenter, and that awareness of such incongruences is crucial to understand collected data and the ability to infer underlying mechanisms. For example, whether the misperception of a GS as the initial conditioned stimulus amplified fear responses and broadened generalization gradients was conditional on the perceptual accuracy during CS trials. In line with Struyf and colleagues (2015), we found that participants with a relative high accuracy on CS trials (cluster 1) adopted an (almost) dichotomous response strategy. Stimuli classified as CS elicited a strong fear response whereas the same stimuli classified as different from the CS were related with an attenuated fear response. For participants that made many errors on CS trials, the perceptual categorization of a stimulus as the conditioned fear stimulus or as a different stimulus did not affect the strength of fear responding. As the US is presented (during the generalization phase) ignorant of perception, perceptual errors on those trials will result in another learning experience: not only the CS but also other stimuli (GSs) predict the US. Hence, whether a stimulus is perceived as the CS or a GS should not affect the strength of fear response as both are perceived as threatening in the experience of the actor. Surprisingly, we only found such relationship for US expectancy ratings. For startle eye blink potentiation, the perceptual categorization of a stimulus related to the strength of responding while Cluster allocation did not relate to this response nor interacted with the effect of perceptual categorization. The lack of convergence between this index of fear learning and US expectancy ratings, fits with the notion that these measures represent distinct dimensions of learning^18,19^ with startle responding independent of explicit awareness^20,21^. Future studies should investigate the role of perception in this context in more depth.
